# Transactions of the Tri-State Medical Association

**Published:** 1895-12

**Authors:** 


### Society Reports.

### TRANSACTIONS OF THE TRI-STATE MEDICAL ASSOCIATION.

(*Concluded.*)

A. R. ROBINSON, of New York, read a paper on

### THE TREATMENT OF MALIGNANT CUTANEOUS EPITHELIOMATA (CANCERS).

In cases where the diagnosis is not positive, iodide and mercury should be used. The elements in these cases extend much farther than is generally supposed. When they are cut out pathological cells are left, and we have so-called recurrence, which is a reappearance. When the wound has been treated antiseptically, it should be allowed to suppurate, as the toxine of the pus is more destructive of the epithelioma cells than the erysipelas toxine. He opposed cutting and advocated caustics. The toxines had cured no cases, but some may have been benefited. The caustic should destroy the tissue completely and quickly. Mild caustics, as nitrate of silver, should not be used.

Caustic potash liquifies the tissue as much as would be removed with the knife; beyond this there is an inflammatory exudation which destroys the pathological elements. The cancer cells may extend deeper than could be reached with the knife; here, the potash is preferable. There is less deformity than with the knife, more than with arsenious acid.

Chloride of zinc acts more slowly, suitable only in certain locations, as near the eye; also, in the papillary form, previous to the use of arsenious acid. Pain may be avoided by mixing in twenty per cent. cocaine solution.

Arsenious acid has a more elective action on the cancer cells and should, as a rule, be used weaker than Marsden's paste (2 to 1 part of gum arabic). It may be used 3 to 2 or 1 to 1. This will not attack normal tissue in twenty hours. It should be removed, and if not sufficient necrosis, applied immediately for sixteen to eighteen hours. Cases should be watched for a year or two, as there may be a reappearance. Arsenious acid may be applied to the lip, if care is taken to prevent getting it into the mouth, but on the nose, it is especially valuable on account of deformity.

These papers were discussed together.

R. R. KIME said that it was unfortunate that cancer of the uterus was not diagnosed early. The symptoms were sometimes not well defined, even when the disease was so far advanced that an operation is not advisable.

Cases from thirty-five to fifty should be examined, if any suspicion of cancer. If the general practitioner is in doubt, he should refer the case to the gynæcologist. If confined to the cervix, this may be removed, followed by caustics; the fatal results of hysterectomy being so much greater than in amputation of the cervix.

W. E. B. DAVIS thought that these were overlooked as they developed so insidiously. Every case of diseased cervix should be treated and cured. He advised removal of the uterus and ovaries for cancer.

P. L. BROUILLETTE asked as to the danger of systemic effect from arsenious acid.

DR. GOGGANS said that wherever there was cancer, there had been irritation, hence the necessity of removing every source of irritation and every pathological condition as prophylactic.

DR. ROBINSON had applied arsenious acid on a surface as large as the hand and had seen no systematic effect. Marsden advised not over an inch square. If seen early, caustics are all that are necessary in cancers of the skin.

J. P STEWART read a paper on

### HEMORRHOIDS, in which he dwelt on the necessity of an examination under chloroform, if necessary. Where tumors are small, he used an injection of red gum; if larger, they are destroyed with clamp and cautery.

R. P. JOHNSON had treated cases successfully with injections of carbolic acid, 2 parts to 1 of olive oil, to which was added one-fourth grain of morphine to each injection.

J. B. COWAN alluded to the new operation of excising all the hemorrhoidal tissue. These cases often fall into the hands of quacks, who inject carbolic acid or ergot and temporarily relieve them. He likes the old method of tying the tumors. In a bad case, where there was no distinct tumor, he applied a method which was successful. This was to take up the tissue with tenaculum and tying a part at a time. He thus put in fourteen ligatures. The result perfect.

R. R. KIME dilates the sphincter and dissects the membrane from the tumor, which he ties, if large. He called especial attention to the value of the knee chest position, thus ballooning the rectum.

G. A. BAXTER insisted on not confining the treatment to any one method. Carbolic acid, he considered dangerous. Could not examine without paralyzing the sphincter. He was always able to find a distinct tumor.

J. B. MURFREE said it was better to dissect up the mucous membrane and ligate the vessels (after Allingham) than the whole tumor. Carbolic acid valuable as palliative, and safe as a rule.

R. P. JOHNSON said that the piles are destroyed with carbolic acid, if the solution is strong enough and the piles are filled.

DR. STEWART said that carbolic acid was a complete failure.

R. R. KIME read a paper on

### SYNTHETIC PERINEOTOMY IN LACERATIONS OF THE PERINEUM, using the term to designate a method of dividing and dissecting without loss of tissue. The redundancy is due to hyperplasia, a subinvolution which will disappear when the cause (the laceration) is removed. The method was described in detail and cases related.

W. E. B. DAVIS said that there was more in the man being familiar with the operation he practiced than in any particular operation. It was important to differentiate between perineal tears and those of the posterior vaginal wall.

W. E. B. DAVIS, of Birmingham, read a paper on

### BILE IN THE PERITONEAL CAVITY AND HOW TO DEAL WITH IT.

He presented an experimental and clinical study of the subject. The experiments confirmed the position taken in 1892 before the American Medical Association. The constant extravasation produced peritonitis unless there was satisfactory drainage. A considerable quantity would be walled off just as any other irritating fluid. It was noted that in those cases where gauze was packed around the openings in the gall bladder or ducts, that the animals recovered, as a rule. The field of operation was walled off completely. Numbers were reopened in twenty-four to forty-eight hours and this condition found. In an operation on the human subject in which the gall bladder was removed and drainage with gauze and a glass tube, the field of the operation was completely walled off and there was no evidence of general peritonitis. He took the position that in obstruction of the common duct for stone that an incision should be made, the obstruction removed and drainage established, without an attempt being made to suture the opening, as these cases will not stand long operations, as a rule.

R. J. TRIPPE, of Chattanooga, reported several cases of

### APPENDICITIS, illustrating the necessity of early operation, and the fact that some will die, no matter when operated on, and also gave the technique.

These two papers were discussed together.

R. R. KIME said that authors differed as to time of operation. Many would get well anyhow, but it was difficult to tell which. Where patient was improving and got suddenly worse, operation is indicated, as it is probable that extravasation has taken place. When there is pus in the peritoneal cavity, most operators advise against washing it out, but he would feel safer to flush the cavity and then drain.

C. HOLTZCLAW had had fifteen or sixteen cases of appendicitis but had not been called on to operate. They recovered with the ice pack over the inflamed area. Operation should be performed, if any evidence of suppuration, elevation of temperature, or collapse.

G. A. BAXTER said that danger was not in operating, but in delay. The question lies in the diagnosis between obstructive and catarrhal appendicitis. When we have had a distinct enlargement and induration, we have obstruction.

R. M. CUNNINGHAM said that Dr. Davis was departing from his usual teaching, as he said that normal bile would not produce peritonitis. In a large number of post-mortems, the appendix was not found diseased one time in a hundred. Operation is indicated where there is pus, swelling and induration.

J. P. STEWART always cures his cases of appendicitis with salines. He related a case of injury to the gall duct, followed by distension of the gall bladder. He aspirated and got five pints of pus and bile; a few days later, two pints; and again, one pint; then the obstruction gave way and the same fluid passed per rectum.

J. B. MURFREE said that no operation should be made for appendicitis until some indication, such as recurrence or gross local changes.

P. D. SIMS could not recall a case of death from appendicitis which had not been operated on. It might be required, but swelling and hardness did not indicate an operation.

R. H. HAYES praised the high position taken by Dr. Davis in the surgery of the duct, and asked as to the preparatory and after treatment.

In regard to appendicitis, Telamon, of Paris, had published statistics, giving ninety to ninety-five per cent. of cures without operation. An eminent English author gave out similar statistics. Whether these are reliable or not, he does not know. Many claim that when one has an attack of appendicitis, he has a constant source of danger in his belly. A few years back, this was the main indication of an operation, and in this view, it seemed that the operation should be performed at any opportune time.

DR. DAVIS thought that the case of Dr. Stewart was not a distension of the bladder, but that the cyst had formed around the bladder. It was saved by the aspiration. The after treatment was the same as for other abdominal sections.

He thought catarrhal cases of appendicitis were common. When there is obstruction, there is pain. Each case should be treated on its own merits. There are no hard and fast rules. Some needed operation. In general septic peritonitis the patient will die if operated on.

THIRD DAY.

J. R. RATHMELL, of Chattanooga, read a paper entitled

ACROMEGALY; REPORT OF CASE.

He said that Paul Marie first described disease in 1886, his theory being that enlargement of the pituitary gland was the cause of the disease. In the case reported, with the symptoms common to this disorder, there were two uncommon symptoms. These were long continued abnormal rhythm of the Cheyne-Stokes variety and the inability to retain food or drink on his stomach for three months before. Both symptoms were accounted for by the enlarged pituitary body, which weighed 475 grains, instead of 5 to 10 normally. The author believes the disease to be one of trophic origin, producing changes in the bony system, especially of the face, feet, and hands; that the enlargement of the pituitary body and the other ductless glands was the result, not the cause of the disease.

W. C. TOWNES, of Chattanooga, read a paper on

ACROMEGALY, exhibiting specimens from Dr. Rathmell's case—the enlarged pituitary body and part of the ileum, with a diverticulum. He reported a case now under treatment, a marked feature of which was that although fifty-two years old there was no impairment of the sexual function. He believed the disease due to pressure on the brain produced by the enlarged pituitary body.

W. G. BOGART said these cases were rare. He had seen Dr. Rathmell's case. The enlarged tongue made articulation difficult. The breathing was labored and gave evidence of suffering. The points of interest were the length of time required for the development of the case, fourteen years, and the question whether anything can be done if the diagnosis were made early.

E. A. COBLEIGH asked if it were not possible that the condition was a persistent accentuation of a normal process, and if so, as to the cause. He reported a case presenting some of the symptoms.

J. BERRIEN LINDSLEY said that the paper was an evidence that the profession was advancing, and in this respect second to none. A disorder was being discussed the name of which had not yet gotten into the dictionaries.

J. R. RATHMELL said that the first evidence of the disease was loss of strength and enlargement of extremities. He believes it a disease of the osseous system, especially of the feet, face and hands, but it affects the whole bony system, just as we have a pseudo-hypertrophic muscular paralysis. The line of treatment was to build up the system, and electricity. He got some comfort from the latter.

J. B. COWAN, of Tullahoma, Tenn., read a paper on

### WATER VERSUS ATMOSPHERE—CAUSE OF MALIGNANT MALARIAL FEVER, and related observations where water from clear springs was evidently the cause of malarial fever. One case where there were two springs connected underground; the upper was healthful, while those who used the lower had the disease. A marsh between the two formed a favorable nidus for the development of the miasm. In almost every instance where well and spring water had been abandoned for cistern water, malarial fever had disappeared. In a village supplied by a well all had the disease, except one family that used other water. So many similar instances had come under observation that he invariably changed the water in his malarial cases. If sterilized water were used, malarial fever would be unknown.

E. T. CAMP could not agree that malaria was taken in the water alone. Some of the poison might be so absorbed, but it was said that in the tropics one would get a chill by sleeping in the open air. He believed it was taken mostly through the air.

C. HOLTZCLAW differed from Dr. Cowan and related a case where there was no improvement by removing to a location where there had been no malaria for eight years.

W. G. BOGART said that water was one of the means of introducing the poison into the system, but not the only means. In a certain neighborhood where different waters were used all suffered alike, whether they were cleanly or not.

Y. L. ABERNATHY observed that when we have a wet summer, we do not have much malarial fever. The paper seems conclusive that we can get the disorder from water, but why don't we have it every year, and why don't we have it all the time? The old theory was that it was due to heat, moisture and decomposition. Bowling's theory was that it was due to water confined so as to prevent evaporation. Now, it is said to be due to a germ. Whichever is correct, it is true that nineteen-twentieths of the diseases of the West and South are due to malaria, so that we have to give quinine, iron and arsenic, etc.

Dr. COWAN said that the reason we did not have the disease every summer was because the heat did not develop the germ. All cases of malignant malarial fever were due to drinking water. In parts of the South where formerly prevalent and now unknown it was due to the use of cisterns. In one case, a party camped several years on a lake and had malaria; they escaped by taking their water with them, although sleeping in the open air as before.

E. T. CAMP, of Gadsden, Alabama, read a paper on

### A COMPLICATED CASE OF OBSTETRICS, WITH RUPTURE OF THE UTERUS.

It was a transverse presentation. The child was asphyxiated, but resuscitated. The placenta not being delivered in an hour, choloform was administered, the hand introduced into the uterus, when the rupture was discovered. The placenta was adherent, but was torn away; morphine was administered, but death took place in four hours. He thought rupture took place before the version and was spontaneous. He thought if the placenta could have been gotten away and contraction induced, the patient might have been saved.

Y. L. ABERNATHY thought this possible if a laparotomy had been performed.

W. G. BOGART said that the three interesting points were, first, the time of the rupture; second, the cause; third, the ab sence of symptoms. We would expect to have shock, with all the well-marked conditions following. The strange thing is that there was no pain, loss of blood or shock. With the complications—mal position, adherent placenta, and rupture of the uterus, with bad surroundings—death was inevitable.

J. P. STEWART thought the patient died from hemorrhage, not from shock. A post-mortem would have been of interest.

D. S. MIDDLETON thought the adherent placenta indicated a diseased structure, and this caused the rupture. The administration of chloroform opened the mouths of the vessels, and death was from hemorrhage.

C. HOLTZCLAW said that the only chance was to sew up the rent after doing a laparotomy.

J. R. RATHMELL said that having a general adherent fibroid placenta, the only thing to do was to let it alone and remove the entire uterus.

G. A. BAXTER thought that an operation should have been done quickly, and the operator governed by circumstances, after opening the abdomen.

R. M. CUNNINGHAM thought the doctor did the best to conserve his own interest. The case would have died any way. To be in line with modern surgery, an operation should have been performed.

DR. CAMP said that there was nothing to indicate the condition until discovery was made. In a similar case he would not operate, as the adherent placenta would have produced death later.

R. H. HAYES, Union Springs, Alabama, read a paper on

### NUCLEINS AND THEIR RELATIVE POSITION IN THERAPEUTICS.

The nucleins are protoplastic or bioplastic cell substance, the bioplastic primal unit of the organism; the cell-life, vital and resistant force; a proteid, granular, cell-life substance, in which is all vital energy and cell-life resistant force, and through which all animal nutrition took place. The nucleins are proteid bodies residing in the tissue cells and the yeast of certain plants (animal and yeast nucleins). The former taken from the blood and lymphoid glands of the body, residing principally in the polynuclear blood corpuscles or leucocytes, the proliferation of which they have the power of increasing (leucocytosis) They are natural defenders, arresting and overwhelming all alien or disease germs as they enter the blood stream. Vaughan and McClintock have developed them.

They are gotten up in three forms, nuclein solution from the yeast of certain plants (vegetable); nuclein solution from the tissues of the body, thymus, thyroid, liver, spleen, etc. (animal); and from the tissues direct (protonuclein). The principal difference between the antitoxines and toxines is that the toxines antidote or antagonize a poison or ptomaine formed by the presence of alien or disease germs, and they belong to the class of albumen serums and attack the germs direct as soon as they reach the blood current. The nucleins are more direct, if less powerful, and have the advantage of attacking through the leucocytes any or all germs or poisons entering the system. Reports are encouraging from eminent men. The author reported ulcer of sixteen years standing, cured in four months. Another case of ulcer of the ankle (both non-tubercular) very greatly relieved in same time. He favors, from limited experience, more general application of the nucleins.

G. W. DRAKE endorsed the use of these agents on theoretical grounds and intends to use them and await results.

C. HOLTZCLAW cited the transfusion of blood as being a use of nucleins; salt water does as well. The Valentine meat juice injected near the ulcer was found beneficial, but water does better. The whole business was an advertising scheme. These things should be under the control of the government.

R. P. JONES read a paper on

### TREATMENT OF DIPHTHERIA, and gave his experience with established methods, endorsing especially the benzoate of soda. He read a letter from Professor Klebs, who did not speak favorable of antitoxine, but claimed better results with antidiphtherine. He presented a plan to steam the patient with quicklime by covering him with a sheet to loosen the membrane.

G. A. BAXTER said that as the danger was from sepsis, and the germs were under the membrane, they could not be reached by local treatment.

In a case treated here, the membrane liquified quickly under the serum treatment. Where death was from obstruction which might extend to all parts of the lungs, no agent known would liquify quickly enough.

GEORGE THRASH said that Professor Klebs, in a paper read before the profession at Asheville, did not seem to be very enthusiastic about the serum treatment.

Y. L. ABERNATHY said that there were cases so mild as to need no doctor, and some so bad as to need no doctor. The membrane is not dangerous until it gets into the trachea, and then the case generally dies. Trachæotomy is all that promises anything, and it is generally a failure.

J. BERRIEN LINDSLEY presented a paper on the

### PREVENTION OF SMALLPOX.

He did not believe one-fourth of the people of Tennessee were vaccinated. Doctors were not willing to acknowledge their inability to diagnose smallpox, hence, mistakes occur. In Little Rock, the disease was under way six weeks before its nature was discovered. A rule of sanitary boards is, in case of doubt, to give the public the benefit of the doubt and isolate the case. All suspicious cases should be sent to some institution.

He related several cases where undiagnosed smallpox spread, resulting in death.

H. BERLIN said the micro-organism of the disease had not been discovered, and we were left to empiricism. Jenner had done more for the race than Napoleon in all his wars. In the Franco-Prussian war, the French lost more from smallpox than were killed in battle; smallpox was unknown in the German army, as the soldiers had been vaccinated.

R. P. JOHNSON did not think the disease very contagious until pustules were formed. Danger increases with desquamation. Physicians should have their patrons vaccinated.

DR. LINDSLEY said that the reason smallpox was dangerous was because of the neglect of vaccination. The fear of it did not formerly exist.

The profession had not done its duty in the matter, failing to insist on its importance.

The following officers were elected for the ensuing year:

President, J. B. Murfree, Murfreesboro, Tennessee; Vice Presidents, R. J. Trippe, Chattanooga; R. R. Kime, Atlanta; R. H. Hayes, Union City, Alabama; Secretary, Frank Trester Smith, Chattanooga; Treasurer, G. R. West.

The next meeting will be held in Nashville, October 13th, 14th, and 15th, 1896.

The following were read by title:

"When Consumptives Should Go to Colorado, and Why," J. C. Minor; "Diagnosis of Incipient Phthisis," L. P. Barbour; "Some Simple Procedures in Tedious Labor," R. M. Harbin; "Sympathetic Ophthalmia," Frank Trester Smith.

